# Book Reviews

**Published:** 1829-09

**Authors:** 


### CRITICAL ANALYSES.

Quæ laudanda forent, et quæ culpanda, vicissim

Illa, prius, cretâ; mox hæc, carbone, *notamus.*—Persius.

*A Practical Synopsis of Cutaneous Diseases, according to the Arrangement of* Dr. Willan; *exhibiting a concise View of the Diagnostic Symptoms and the Method of Treatment.* By Thomas Bateman, m.d. f.l.s., Physician to the Public Dispensary, and Consulting Physician to the Fever Institution. *The Seventh Edition.* Edited by Anthony Todd Thomson, m.d. f.l.s., Member of the Royal College of Physicians, and Professor of Materia Medica and Pharmacy in the University of London, &c. &c.—8vo. pp. 460. Longman, London, 1829.

Dr. Bateman's work on Cutaneous Diseases is too generally known to require at the present time any abstract of its contents, or any opinion of its merit. Its character as an excellent practical book is firmly established in the estimation of the profession. We shall, therefore, confine ourselves principally to the improvements and additions which have been made by Dr. Thomson, as editor.

The arrangement of the diseases described remains the same as that which was adopted by Dr. Bateman, "from a desire that the work should still retain the stamp and impression given to it by its excellent author." To render the work as useful as possible to the student, has been the chief object of the editor. With this view he has added the synonyms of each genus and species; and, by giving the definitions in a distinct form, he has impressed on the whole a more definite character. He has been enabled also to add considerably to the practical part of the work, from the opportunities afforded to him by his connexion with two extensive medical charitable institutions. Much to the advantage of the student, also, Dr. Thomson has added, at the close of each genus, a list of the works which may be consulted on the diseases that constitute the genera.

No verbal description of cutaneous diseases, however lucid and accurate it may be, can convey to the mind a sufficient idea of their appearance. In this branch of medical study, plates are absolutely necessary, and the delineations of the author of the Synopsis are admirably calculated to fulfil this object, but their expense places them beyond the reach of most students. To remedy this objection, an atlas of plates is published with the present edition. It contains almost all that really relates to the diseases delineated in Dr. Bateman's plates, with the addition of many original representations. To give them the character of demonstrations, the different stages of the eruptions, and other peculiarities necessary to be pointed out, are marked upon the plates: their convenience and utility as references are thus greatly enhanced.

Dr. Thomson gives a brief sketch of the life and character of Dr. Bateman, the materials of which were furnished by the "Life" written by his sister. His professional character is thus favorably portrayed:

"Upon the whole, it may be truly said of the author of the Synopsis, that few men have achieved so much in so short a course of years; that he sacrificed his life to the love of his profession; and that he has deservedly merited a place among the honoured few whose exertions have contributed to dispel the clouds which so long obscured the light of science from the paths of practical medicine."

It is evident that each chapter of the former edition of the "Synopsis" has been very carefully revised, and most of the subjects that are discussed have been more or less improved by the experience of the editor, and by frequent references to additional authorities.

We select a few of the practical suggestions for which we are indebted to Dr. Thomson.

Upon the subject of *Lichen agrius,* he observes that "sulphureous baths, which are undoubtedly useful in several cutaneous affections, invariably increase this form of lichen, unless the disease has become chronic, and is disposed to pass into impetigo. I am aware that this is contrary to the opinion of Mr. Plumbe; who remarks that, after the 'bowels have been for some time kept open,' and the habit reduced, 'the itching and tingling during the operation of the sulphur bath, is rather severe; but it is followed by a much more tranquil state of the circulation in the cutaneous vessels, and the cure is altogether expedited by it.'"*

Dr. Bateman observes, that the causes of *Psoriasis* are nearly as obscure as those of *Lepra.* In almost all the cases which Dr. Thomson has seen, except those of a purely local nature, the digestive organs have been in fault, and great acidity of stomach has prevailed. He thinks it not improbable that the arthritic diathesis, mental anxiety, the unseasonable employment of the cold bath; a copious use

* Practical Treatise on Diseases of the Skin, p. 196.

of acid fruits, vinegar, or crude vegetables; or some peculiar mixtures of food, always produce this state of stomach previous to the appearance of psoriasis; "and it is probable that the irritable state of the stomach, which gives rise to the imperfectly formed gastric juice in these cases, is accompanied by a corresponding irritable condition of the skin, which inducing subacute inflammation of the superficial capillaries, causes the cuticle to be secreted in the diseased state which characterises this eruption."

In *Psoriasis inveterata*, Dr. Thomson informs us, the use of the arsenical solution has in many instances been found highly beneficial, when the dose has been gradually carried to an extent which would be dangerous in other states of the habit.

"Thus, in a case successfully treated by Mr. Gaskoyne, the dose was gradually increased to thirty-eight drops twice a day; and it was not until the desired change occurred in the eruption, that the colicky pains and other symptoms of an overdose of arsenic presented themselves. Candour obliges me to acknowledge that, notwithstanding the powerful influence of arsenic in Psoriasis inveterata, I have met with cases which resisted it, even when administered in the largest doses. In some instances, erysipelas has accompanied the use of the arsenical solution; in which case the administration of the remedy should be suspended until the erysipelas be removed, and afterwards renewed in smaller doses."

"From my own experience, I can confidently assert that the medicine on which the greatest confidence may be placed, in Psoriasis diffusa and in the milder cases of P. inveterata, is the liquor potassæ. I usually commence with thirty drops of the solution in two fluid ounces of the bitter-almond emulsion, twice a day; and gradually increase the dose of the solution to eighty, or even one hundred drops. If the patient be delicate, the infusion of yellow cinchona bark, or of cascarilla, is substituted for the almond emulsion. I have frequently found the hydrargyrum cum cretâ, in doses of six or eight grains, given at bedtime, an useful adjunct to the solution of potash." (P. 67.)

The following interesting case of *Ichthyosis simplex* is also given by the editor:

"The patient was about fifteen years of age in the spring of the year 1810, when the disease was first observed. She had previously been subject to headachs, flatulence, disordered bowels, cold feet, and flushing of the cheeks. The first symptom was little more (to use her own words,) than a soiled appearance of the cheeks, which was easily washed off with warm water and soap; and it was not until the autumn of 1812 that this soiling began to increase and adhere more firmly; and in the course of a few months it became so considerable, that the parents of the patient consulted the late Dr. James Gregory, of Edinburgh. After the use of some acrid applications, which produced inflammation and ulceration, Dr. Gregory succeeded in clearing the skin in ten days. This improvement, however, was of short duration: the disease returned; when steel and aloetics, mercury carried to salivation, warm sea-water baths, shaving off the incrustation, an ointment composed of carbonate of soda, spirit of turpentine, sugar, and resin ointment; a strong lotion with oxymuriate of mercury, and various other means, were successively employed to clear the skin, for three years, but without success; when she came to London for further advice.

"The eruption at this period extended over both cheeks, and across the bridge of the nose: it was of a dirty olive-brown hue, and greatly disfigured a face which was naturally very beautiful. It had much the appearance and the harshness of shagreen. Under Dr. Bateman's care, the patient took pitch pills, and employed various internal and external remedies, but without any permanent benefit, for six months; when, becoming tired of medicine, she resolved to return to Scotland uncured. The editor, however, having persuaded her to remain in the metropolis, after the empirical trial of many remedies, succeeded in completely removing the eruption, by means of a decoction of the root of the sharp-pointed dock, *Rumex acutus*, taken internally. In eight days the skin acquired its natural texture and appearance; but, the use of the decoction having been discontinued, at the end of ten days more the eruption reappeared; it was again removed by the decoction; and in this manner it was combated at successive intervals for several months, always returning a short time after the decoction was discontinued. Conceiving that the return of the eruption depended on a habit acquired by the skin from the long continuance of the disease, the face was blistered with the cantharides plaster immediately after the eruption was again cleared off, and the cure became permanent." (P. 80.)

Dr. Thomson was induced some years since to apply the hydrocyanic acid, in the form of lotion, in *Impetigo:* he found that it allayed the irritation more effectually than any other means. It has since been very generally employed for this purpose. The following is the formula which he originally used:

℞. Acidi Hydrocyanici fʒiv.; Aq. distill. f℥vij.; Alcoholis fʒiv. M. fiat lotio.

Dr. T. has lately found that the efficacy of this application is greatly increased by the addition of sixteen grains of the acetate of lead. This lotion, he says, not only soothes the irritability of the part, but also disposes the skin to take on its healthy action. Mr. Plumbe cautions us against the external employment of hydrocyanic acid, and mentions that in two cases, of both legs, in which the eruption extended from the ankle to the knee, where it was employed, a considerable intermission of the pulse took place, which ceased on its being discontinued. Dr. Thomson has not seen any bad effects result from the external use of this remedy. We have ourselves frequently employed the prussic acid in the form of lotion, particularly in herpetic eruptions, and have found it succeed better than any other remedy in allaying the tormenting tingling and irritation of the diseased surface; but we have never ventured upon a stronger form than a lotion composed of *one* drachm to six ounces of water; and we confess we should *have been* fearful of using it of nearly *four* times that strength, as Dr. Thomson recommends, especially upon an abraded surface, or over broken pustules or vesicles.*

Dr. Bateman observes, that the functions of the stomach, and of the sensorium commune, are not evidently disturbed by that kind of vesicular disease termed *Eczema,* although the extreme pain arising from the pressure of the weight of the body upon an extensive portion of such a raw surface is sufficient to give rise to an acceleration of the pulse and white tongue. The experience of the editor obliges him to dissent from this opinion.

" In almost every case which has come under his notice, there has been evident constitutional derangement, quick pulse, furred tongue, and impaired appetite, with considerable nervous irritability. Indeed, the latter state has been so frequently present as to induce the editor to regard an irritable state of the nervous system, such as produces hysteria in females, to be the predisposing cause of this disease, when it occurs during a mercurial course. The following case will illustrate this opinion:

" A young woman was seduced from her parents, and brought to London, by one of those unprincipled men who sacrifice every moral and social feeling on the altar of self-gratification. Desire and the pleasure of possession having subsided, the wretched victim of unbridled passion was soon deserted, and fell into a course of life which any deviation from the paths of virtue usually produces in the female sex thus situated. She went upon the town, as the term is, and in that wretched and precarious state of life contracted syphilis, for the cure of which she was placed under a course of mercury. Her father, whose paternal feelings were not destroyed by the stain which the misconduct of his child had affixed on the character of his family, had followed her to town, and in vain had endeavoured to discover her retreat. At length

* We have always used the prussic acid made by Mr. Garden, of Oxford street: it is equivalent to Scheele's in strength, although the mode of preparing it is somewhat different.—EDITORS.

he met her in the street, when she was labouring not only under disease, but when her habit was charged with mercury for its relief, and when she was reduced to a state of extreme indigence. Her eye met that of her parent, and she fled as rapidly as she could from an interview which she dreaded; and, although her father closely followed her, yet she secured her retreat to her lodgings for that night. On the following day she was too ill to move from home; her mouth was affected, and the salivation considerable; when late in the evening of that day she heard her father's voice at the door of the house in which she lodged. She instantly left her bed, and escaped into another room as he entered the one in which she had been lying, and ran into the street in a half-naked condition, during a heavy shower of rain. I was requested to see her on the following day. She was then covered in patches with an eruption, which, to the unassisted eye, much resembled that of scarlet fever. She complained of great heat, stiffness, and tingling upon the inner and upper surface of the thigh, and round the neck and waist. In these parts patches of extremely minute vesicles were apparent, gradually extending themselves over the whole body. The stinging and irritation increased to a degree almost insupportable. There was fever, which in a few days assumed an intermittent character; and a very fetid odour exhaled from the body. The viscid fluid which oozed from the patches dried and crusted, and the cuticle peeled off in large pieces. In this state the disease continued for ten days. The warm bath, anodyne fomentations, liniments of linseed oil and lime-water, were externally applied; whilst saline purgatives, refrigerants, decoction of cinchona bark, the mineral acids and opium, were internally administered, without any beneficial result. In fifteen days from the commencement of the attack, the wretched girl died, in a state of suffering which no language can correctly describe.

"In this case, the mental alarm had predisposed the body, under the influence of the mercury, to be excited by the sudden exposure to cold and damp in a peculiar manner; for that it was not cold and damp alone, is probable from the fact that the poor creature had been driven by strong necessity to walk the streets in all states of the weather, during the whole period in which she had been taking mercurials, without suffering, until the nervous system received the shock which has been described." (P. 363.)

A particular kind of *Nævus* is sometimes met with, which, although originally very small, becomes a large and formidable bloody tumor, readily bursting, and pouring out impetuous and alarming hemorrhages, which, if they do not prove suddenly fatal, materially injure the health by the frequent depletion of the system. A striking case of this kind fell under the notice of the editor about ten years since. As the description of it is very interesting in a pathological point of view, we subjoin the account of it.

"The little patient was born without any apparent mark upon the body, nor did any appear for eight days after birth, when a small point, resembling a red minute tubercle, appeared on the forehead, and gradually increased to the size of a crownpiece; when it was showed to the editor. This spot was surrounded by many small points, at different distances from the main spot; and these, gradually enlarging, ran into one another, forming larger spots; which again in turn coalesced with others, until they finally were added, as their diameters increased, to the main spot. (Atlas, pl. xxv.) The extent which the whole occupied, and the eye being also involved in the disease, prevented extirpation from being proposed or attempted; and the only curative measure resorted to was an effort to obliterate the nævus, by exciting ulceration in various parts of it. This partially succeeded; but, before the plan had advanced beyond the second sore, the child was attacked with hydrocephalus, and died. A post-mortem examination explained satisfactorily the nature of the disease. The arterial system was natural, but the venous was so thin in the coats of the vessels, that there was not sufficient power to return the blood, which of course accumulated in the veins; and those in the vicinity gradually assumed the same diseased state. The most remarkable part of the case, and on account of which it is mentioned in this place, was the extension of the disease to the bones of the cranium. The editor is not aware of any case of a similar kind being on record. The skullcap is in the museum of anatomy of Dr. Alexander Monro, of Edinburgh, to whom the editor presented it; and an accurate engraving of it will be found in the atlas of plates attached to this edition of the Synopsis." (P. 448.)

We have seen two cases of nævi, each about the size of a shilling, in which there was slight thickening, elevation, and altered structure of the skin, with an enlargement of the veins of the part. By vaccinating these nævi in several points, they were completely cured. A discoloured spot, of course, remained.

Several cases successfully treated in this manner have been lately published in different journals. The narrator of a case* which occurred in the Glasgow Infirmary observes, that it is indispensable to the ultimate success of the practice that the vaccine lymph should be freely introduced over the diseased surface, as well as around its circumference. In this way the adhesive inflammation which is excited appears to extend from one pustule to another, and in the course of a few days the whole becomes involved in one scab."

* London Medical and Physical Journal, July 1829, p. 80.

The extracts we have given will show that the editor has not merely followed his original. He has added to the practical value of the work in almost every page; but his observations are necessarily so incorporated with the text of Dr. Bateman, that it would be impossible to do full justice to them without entering into a more extensive analysis of the work in general than we deem justifiable, as its contents are well known and duly appreciated.

Dr. Thomson deserves the thanks of the profession for this much improved edition of the "Synopsis of Cutaneous Diseases." He has not only added many practical remarks which were overlooked by Dr. Bateman, but he has also introduced many important suggestions which have arisen from subsequent experience.

The plates of the atlas are elegantly and correctly executed, and are moderate in price. By attentively studying them in connexion with the text, which they so well illustrate, the student cannot fail to gain a competent knowledge of cutaneous diseases.

---

*Elements of Medical Statistics; containing the Substance of the Gulstonian Lectures delivered at the Royal College of Physicians: with numerous Additions, illustrative of the comparative Salubrity, Longevity, Mortality, and prevalence of Diseases, in the principal Countries and Cities of the Civilized World.* By F. Bisset Hawkins, M.D. of Exeter College, Oxford; Fellow of the Royal College of Physicians; and Physician to the Westminster General Dispensary.—8vo. pp. 234. Longman, London, 1829.

Although much has been done towards an illustration of the medical statistics of various countries, cities, towns, and hospitals, by numerous inquirers, it is yet to be lamented that so important a branch of science, one from which our practical knowledge would derive such vast improvement, has not produced any single volume to which reference might be made for the purpose of instituting comparisons respecting the influence of climate or local habits, and the peculiarities of disease, in different parts of the world. It is true, as Dr. Hawkins observes, that a great variety of important single facts has been gradually exhibited, by writers engaged on their own particular topics, and by medical and other journals. Of these, many, from their fugitive form or insulated situation, have been neglected or forgotten; and "Reports," which have been matured with severe labour and disinterested patience, have sometimes appeared valueless, because unaccompanied with the materials of comparison. The author conceives that a favorable moment is, perhaps, at length arrived for arranging these scattered fragments into the rudiments of a system, and for comparing together, in close apposition, the documents afforded by different countries and institutions, which at present lie far asunder. He is perfectly conscious of the difficulties and dangers of the subject; of the dubious authenticity and frequent fluctuation of the necessary details; and of the precarious nature of any general principles attempted to be framed out of facts which have, for the most part, only endured the test of a few years, and which have only recently become the object of inquiry or scrutiny.

An extensive assemblage and classification of such facts possess an historical and local value, whatever may be the fate of the reasonings deduced from them. Independently of the light which this study throws upon medical science, it affords the most valuable illustrations of the history, manners, and customs of mankind, and a just criterion of the progressive or retrograde movements of society.

Dr. Hawkins is not aware of the existence of any work in the literature of Europe which treats the subject in all its parts, or which takes so extensive a range as the present; a circumstance which, he trusts, will form the best apology for any inaccuracies or omissions inseparable from a *first* attempt to sketch the outlines of a system.

Chapter i. *Utility and history of the subject; comparison between the value of life in ancient and modern times.*—The word "statistics" appears to have been first used about the middle of the last century, by Achenwal, a professor at Göttingen, to express a summary view of the physical, moral, and political condition of states. Many important facts which belong to the domain of statistics had been published long before this appellation had been applied to them.

"But some of the details, thus collected for general purposes, were found to throw light upon health and disease; and, on the other hand, it was often necessary to have recourse to medical authorities, in order to elucidate various points of the general picture. A combination of these scattered features forms *medical statistics,* an elementary specimen of which it is the object of the following pages to present. We may perhaps define it, in a few words, to be the application of numbers to illustrate the natural history of man in health and disease." (P. 1.)

The *probability* of life, and the *mean* life, are two expressions which often occur in such inquiries. By the *probable*

life is understood the age to which one half of all who are born in a particular country or city attain. The *mean* life implies the result of adding together the number of years attained by a given number of persons, and of dividing the sum total among each of them in equal proportions.

"Statistics has become the key to several sciences, opening in a manner the most convincing, simple, and summary, their gradual progress, their actual condition, their relations to each other, the success which they have attained, or the deficiencies which remain to be supplied. Its application to the objects of government has created political economy; and there is reason to believe that a careful cultivation of it, in reference to the natural history of man in health and disease, would materially assist the completion of a philosophy of medicine, by pointing out to the physicians of every part of the world the comparative merits of various modes of practice, the history of disease in different ages and countries, the increase and decrease of particular maladies; the tendency of certain situations, professions, and modes of life, to protect or to expose; and by indicating, as the basis of prognosis, those extended tabular views of the duration and termination of diseases, which are furnished at successive periods by hospitals and civic registers." (P. 2.)

Medical statistics affords the most convincing proofs of the efficacy of medicine:* it is one of the easiest arguments that can be employed to refute the vulgar notion that nature is alone sufficient for the cure of disease, and that art as frequently impedes as it accelerates her course. The powers of self-restoration are in no diseases more conspicuous than in fever.

"But, if we form a statistical comparison of fever treated by art with the results of fever consigned to the care of nature, we shall derive an indisputable conclusion in favor of our profession. Hippocrates has left a frank and explicit statement of the history and fate of forty-two cases of acute disease, in which it does not seem that any therapeutical plan was adopted, if we except clysters and suppositories in a few, and bloodletting in one. Amongst these were thirty-seven cases of continued fever, without local affection. Of the thirty-seven, twenty-one died, above half of the whole. But, if we examine the returns of the Fever Hospital of London, we find (in 1825) that the total mortality was less than one in seven; and half of these deaths occurred within seventy-two hours of the admission of the patients, a circumstance which indicates that several entered at a period of disease when the hope of recovery was extinct. In the Dublin Fever Hospital we find a

* The writer in the "Morning Herald," who has lately indulged in such violent, ridiculous, and unfounded tirades against the whole of the medical profession, would do well to dip into the science.—Rev.

still lower mortality: the average from 1804 to 1812 was one in twelve; and in the clinical wards at Edinburgh, in 1818, the mortality of fever was also about one in twelve. Of five cases of local inflammations which Hippocrates records, four were fatal. Of all his forty-two patients, in short, twenty-five were lost; a termination which throws no shade over his skill, but only brings to light his love of truth. The mortality belonged to the age, and not to the physician; and we may reasonably infer that, under other practitioners of his time and country, it was even more severe. It is curious to observe that, of the five cases of local inflammation, the only one which survived was the solitary instance in which bleeding was employed, a pleurisy. We perceive that one out of two acute cases may recover by the almost unassisted efforts of nature, but that, under the medical protection of our own age and country, six out of seven, or even eleven out of twelve, are likely to survive, according to the period of the disease at which they are placed under treatment." (P. 3.)

Medical statistics alone enables us to form an estimate of the influence of various mechanical improvements on the air of certain districts. The town of Portsmouth, for instance, is built upon a low portion of the marshy island of Portsea. It was formerly very subject to intermittent fever; but, since it was paved and drained in 1769, this disorder has no longer prevailed; while Hilsea, and other parts of the island of Portsea, retained the aguish disposition until 1793, when a drainage was made, which subdued its force.

In almost every civilized country of Europe we find every succeeding ten years produce a smaller annual proportion of deaths; and "in Britain the value of life is nearly doubled, if we compare Büsching's rate of one in thirty-two with the actual rate afforded in 1821, of about one in sixty." But the decline in the mortality is even more remarkable in our cities than in the rural districts.

"While the metropolis has extended itself in all directions, and multiplied its inhabitants to an enormous amount; or, in other words, while the seeming sources of its unhealthiness have been largely augmented, it has actually become more friendly to health. Not only its comparative mortality is greatly diminished within the last half century, but its absolute mortality in respect to preceding centuries. In the year 1697, for example, the total deaths were about 21,000; whereas, a hundred years after, in 1797, the amount was only 17,000; and, when we consider the great increase of the inhabitants of the outparishes at the latter period, the change in the health of London will be seen in a powerful light. But it is singular that this healthy condition seems to have been particularly produced within the last fifty or sixty years; during the very period in which it has most rapidly enlarged its limits and its population. In the middle of last century, the annual mortality was one in twenty; it is now (or by the census of 1821) about one in forty. So that, in the space of seventy years, the chances of existence are exactly doubled in London; which is a progress and final result without a parallel in the history of any other age or country." (P. 18.)

In the peculiar circumstances of Ireland, it would be very interesting to know the average mortality; but, unfortunately, no correct parochial registers have been kept.

In Chapter iii. the author shows the superior salubrity of Great Britain, by a general comparison with other countries. On the continent of Europe, we find that changes in the duration of life have been experienced, similar in nature, and following the same laws, as those of our own country, but very inferior in degree.

"Since the late peace, the principal governments of Europe have paid much attention to statistics, and we possess very instructive returns from nearly all the countries, cities, and hospitals on the continent. A comparison of these results enables us to submit a very interesting conclusion, and one which we are not aware to have been as yet generally received, namely, that the mortality of Great Britain, its cities, and its hospitals, is greatly inferior to that of any other country in Europe; and that it is incontestible that Great Britain is at present the most healthy country with which we are acquainted; and that it has been gradually tending to that point for the last fifty years." (P. 30.)

In the comparisons which Dr. Hawkins has made for the purpose of supporting this assertion, he has confined himself to the most recent and genuine details. It is remarkable that this superior value of life in Great Britain is not confined to any particular districts or classes of individuals.

"It has been long the fashion, both abroad and at home, to exhaust every variety of reproach on the climate of our country, and particularly on the atmosphere of London; and yet we shall find that the most favored spots in Europe, the places which have long been selected as the resort of invalids and the fountains of health, are far more fatal to life than even this great metropolis." (P. 31.)

The country which approaches most nearly to us is the Pays de Vaud, where the mortality is one in forty-nine. The annual proportion of deaths at Montpelier was greater thirty years ago, and is greater at present, than in London; and,

"although the mortality of great cities is usually much larger than that of provinces or counties, yet the mortality of London is exactly the same at present as that of the department of the Herault, the southern and fertile, and long-supposed most salubrious, district of France, of which Montpelier is the capital. Finke, a German writer who wrote on medical geography in 1792, speaks with surprise and reprobation of the custom which then prevailed in England of sending invalids to the south of France; and declares that the cutting winds of those quarters annually destroyed many of those wanderers in quest of a milder sky." (P. 31.)

In the next two chapters we have brief, yet interesting, sketches of the medical statistics of various countries and cities.

Chap. vi. *Medical statistics of general hospitals.*—Although it is true that the principal object of hospitals is the relief of the sick poor, another benefit may be derived from them, an abstract of their multiplied experience; without which their utility, as a source of information to our profession, is greatly abridged. Such reports not only tend to improve the economical arrangement of hospitals, but they also collect and accumulate a store of evidence on the history of disease, which can scarcely be acquired in the most extensive private practice.

"Next to the influence of national causes, the mortality of hospitals is most affected by position and internal economy. These circumstances appear more powerful than even the various merits of practice; and, happily for mankind, they are advantages of a definite nature, easily comprehended, and of late years generally demanded. The case was formerly very different, when a singular prejudice or indifference existed in respect to ventilation. At the Leeds hospital, no case of compound fracture nor of trepan survived. At the Hôtel Dieu of Paris, compound fractures were almost always fatal, and few survived amputation. The system which will bear improper air with impunity during health becomes keenly susceptible of its mischief when diseased, and a change of air will often restore where the strictest diet has failed." (P. 77.)

Mortality, says Dr. Hawkins, is *seldom* to be assigned to the influence of bad practice, which probably does not often *destroy* life. From a comparison instituted between the mortality under three physicians in the same hospital, of whom one was *expectant*, one *tonic*, and one *eclectic*, it was found that the mortality was the same; but the length of the disorder, the character of the convalescence, and the chances of relapse, were very different. Moliere, then, it appears, has been too hard upon the medical tribe; for we rarely kill, although we often fail to take the shortest road towards the completion of the cure.

In this chapter, annual and other reports are given from different English and foreign hospitals, which will be found very useful to the medical statist. In the great general hospital, the Charité, at Berlin, which can contain 1000

patients, many are received who pay a small sum for separate rooms and superior accommodation; "a plan which is also encouraged at the great hospital of Vienna, and appears to deserve imitation in our own cities, particularly where the funds of an hospital are not of a permanent nature."

*Statistics of the stillborn*:

"It is scarcely necessary to prove that abortions and still-births are far more frequent amongst the unmarried than among married women. If we observe what happens among the most unfortunate of the former, as in the Hopital des Vénériens at Paris, the excessive proportion of two children out of seven are born dead; and in a similar establishment at Hamburg, the proportion is one in three. If we take a whole town, as Gottingen, only three per cent. of the children born in marriage are stillborn, but so many as fifteen per cent. of those born out of wedlock." (P. 125.)

The statistics of foundling hospitals, and of the diseases of children, and the remarkable diminution which has gradually occurred in the mortality of infancy, form the subjects of the eighth chapter. Whatever may be thought of the *policy* of foundling hospitals, the feeling which created them can only excite respect. Our author cannot help agreeing with Malthus, Beck, and others, that their utility, under any system of *indiscriminate* admission, is highly questionable. It is shown, from various reports from several of such institutions, that they have done very little towards the preservation of infant life; and it is urged that the facilities which they afford corrupt the maternal instinct, and offer a premium to seduction. The foundling hospital of London deserves priority of mention, not merely on account of its excellent economy and the good health of its inmates, but from its standing alone in the principle of rejecting *secret* or *indiscriminate* entries.

"In every hospital where foundlings are indiscriminately received, the mortality appears to be beyond the control of all attention or skill. In Paris, at present, of 1000 foundlings admitted, 251 are ascertained to die during the first few days, and 235 more on their road to the country nurses, or before the end of the first year; so that at that period only half remain alive. It seems that the frail tenure by which an infant holds its life will not allow of a remitted attention, even for a few hours; and that the desertion of a child by its mother, at the very time when, of all others, it stands most in need of her care, is in the event nearly equivalent to its destruction." (P. 132.)

The ninth chapter contains a few brief remarks upon the different asylums for the insane. Upon this subject the excellent work of Dr. Burrows will be consulted with much advantage.

In the chapter upon the increase and decrease of diseases in different countries and cities, we have the following gratifying paragraph: "At the close of the last century, the deaths from consumption had gradually increased from about fifteen per cent. to twenty-six per cent. of the total mortality. From 1799 to 1808 they still increased, being then above twenty-seven per cent. From 1808 to 1818 they, however, declined to twenty-three per cent.; and from 1818 to 1825 they have become still less numerous, being at length only twenty-two per cent., nearly the same proportion as at Paris. At Vienna, it is about seventeen per cent."

Chap. xiv. "*Influence of various conditions, professions, and modes of life, on longevity. Average quantity of disease attendant on particular pursuits.*"—The comparative mortality and longevity of the various classes of society seem to have been formerly balanced by conjecture alone, and it appears to have been even a prevalent opinion that poverty was favorable to long life; that it exempted from numerous diseases which follow in the train of luxury and wealth; and that the affluent individual, if desirous of attaining to old age, would find it his interest to imitate the habits or diet of the peasant.

"The contrary has been brought to light during the present century by a rich variety of facts; and the present conclusion is, that, in general terms, poverty, cold, and moisture, (which two latter circumstances are generally included in the first,) are the greatest enemies to the enjoyment of health and long life, and that competence, or an easy condition, is the strongest safeguard of the body. Of an equal number of infants taken among the poor and the easy classes, it will be found, at least in France, (where the argument has been the most agitated,) that the proportion of deaths among the former is double; and that, wherever is the greatest portion of misery, there will also attend the largest share of mortality. In epidemic visitations, the mortality begins and ends with the poorer classes, and on these are their principal ravages exhausted. It seems to be partly on this account that women (at least in England) die in a less frequent proportion, and are longer lived on the average than men, because they are usually more secluded from the conflict of life, are less exposed to vicissitudes of weather and to severe labour. In France, on the contrary, where the women, in every rank, take a more active part in worldly affairs, and where, among the lower orders, they perform a large part of the manual and out-of-door employments, their mortality (on a late average formed during the six years from 1817 to 1823) appears to be nearly the same as that of the men. Buffon had previously observed, that, in most rural districts, the mortality of females was somewhat higher than that of males, on account of toils unsuited to their frame, which they were there compelled to undergo, and which, it may be added, usually imprint on the female peasant of continental Europe the stamp of old age before she has attained the age of forty." (P. 206.)

From the Paris tables of mortality for 1818, it appears that the mortality of women is not greater at the *critical* period of life than at any other, and that it increases at an advanced age. The conservative tendency of an easy condition is strongly marked by the very inferior degree of mortality and of disease which occurs among persons insured at the various life offices.

"On the other hand, let us observe how great is the mortality of man in his lowest state of want and degradation. It was formerly computed that a fifth or sixth part of the negro slaves died annually. The free Africans who serve in our troops have been said to lose annually only three men out of 100, while the slaves were losing seventeen in 100. At present, however, their mortality decreases in proportion to the superior care taken of them: of about 20,000 slaves landed at Rio Janeiro in 1823, only 1400 had died on the voyage; which would still form an enormous proportion for Europeans, but is a happy contrast to the former returns of a slave ship." (P. 209.)

It is a curious fact, that while the epidemic fever raged in Ireland a few years ago, the army suffered comparatively little from it, because the private soldier is better fed, lodged, and clothed, than the peasant of Ireland. The prevalence was nearly twice greater among the inhabitants than among the army.*

"Cultivation of the sciences appears particularly favorable to longevity, in spite of various assertions formerly made to the contrary: it almost seems that the man who labours chiefly with his mind has a fairer prospect of life than one whose body alone is occupied. Franchini has enumerated 104 Italian mathematicians of different epochs: he has ascertained the ages at which seventy of these died, and among the seventy are eighteen who had attained the age of eighty, and two of ninety; and this, too, in a southern climate, which is not generally very favorable to old age. In France, 152 men of science and letters have been taken at random: half the number appear to have cultivated science, and about half to have been devoted to general literature: on adding together the age at which each died, it was found that the average result would be above sixty-nine years for each of the 152 individuals." (P. 211.)

This statement is certainly opposed to the general belief upon the subject, and we are disposed to think that, if a

* BARKER and CHEYNE, Account of the Fever lately epidemical in Ireland. London, 1821.

more extended comparison were drawn between mental and corporeal labourers, the mortality of the former would be much greater than that of the latter, at a comparatively early period of life.

A statement has been lately published of the deaths which occurred among a society of fifty *plumbers.* "During seven years fourteen members have died, all under thirty-six years of age, and through diseases induced by their business. Dr. Alison believes that there is hardly an instance of a *mason* regularly employed in hewing stones at Edinburgh, living free from phthisical symptoms to the age of fifty."

Dr. Hawkins concludes, with Dr. Heberden, junior, that the presence of infectious matter is not alone sufficient to make the plague epidemical, but that some concurrent state of the air and of the human body is likewise necessary; and that our long exemption from this evil is not so much to be attributed to any accidental absence of its exciting causes, as to our own change of manners, our love of cleanliness and ventilation, which have produced amongst us, if not an incapability, at least a great inaptness, any longer to receive it.

Chap. xv. "*Statistics of the Sexes. Comparative fruitfulness of marriage in various countries.*"—Hufeland asserts, from extensive examination, that the relative numbers of the sexes are in all parts of the world the same, namely, twenty-one males to twenty females.

"Some curious facts have been communicated to the French Academy of Sciences by M. Giron de Buzareingues, relative to the inequalities which occur in different departments of France, in the proportion of male and female births. Of course they are not cited here as establishing a general principle: their value must be determined by a series of observations in other places.

"M. Giron has made several experiments on sheep, horses, and birds, which indicate that, when the male is too young, and the female in full vigor, the proportion of female births exceeds that of male, and *vice versâ.* He affirms that, by attention to this circumstance, we may at will produce an excess of males, or of females, in our flocks, studs, and poultry-yards.

"Pursuing these inquiries with regard to the human species, he divides individuals into different classes: the first is composed of persons whose employments tend to develop their bodily powers; the second, of those whose business tends to enervate; the third, of those whose occupations are of a mixed description. He found that, in the first class, the number of male births exceeded the average proportion of male to female births throughout France; that, in the second class, the number of female births exceeded the average proportion of female to male births throughout

France; and that, in the third class, the proportion of male to female births was nearly the same as the average proportion throughout France.

"He arrives at the conclusion that the pursuits of agriculture tend to the increase of the male population, and that the habits of commerce and of manufactures favor an augmentation of the female population." (P. 222.)

Many tabular reports are given of the diseases of various hospitals in different countries, which are highly important and interesting. We could not insert these in our sketch of Dr. Hawkins' work, but it is proper to observe, that they form one of its most important features.*

Dr. Hawkins has offered the present volume as a "first attempt to sketch the outlines of a system" of medical statistics; and, from the industrious research with which he has collected his materials, and the ability with which he has employed them, we hope he will continue his investigations upon so important an inquiry, and that he will, at some future opportunity, favor the profession with a more elaborate work upon the subject.

---

*Observations on the Phrenological Developement of Burke, Hare, and other atrocious Murderers; Measurements of the Heads of the most notorious Thieves, presenting an extensive Series of Facts subversive of Phrenology.* By THOMAS STONE, Esq. Psq. President of the Royal Medical Society, Edinburgh.—Edinburgh, 1829.

*Answer to "Observations on the Phrenological Developement of Burke, Hare, &c. by* THOMAS STONE, Esq." By GEORGE COMBE.—Edinburgh.

*A Rejoinder to the Answer of* GEORGE COMBE, Esq. *to "Observations on the Phrenological Developement of Burke, &c."* By THOMAS STONE, Esq. President Royal Med. Soc. Ed.

*Anti-Phrenology; or, Observations to prove the Fallacy of a modern Doctrine of the Human Mind, called Phrenology.* By JOHN WAYTE, M.D. Lynn-Regis.—Baldwin and Cradock, London.

IT is not to be imagined, from the above pompous display of titles, that we are about to plunge either ourselves or our readers into a formal discussion of the extensive, and we fear inextricable, maze of phrenological perplexities. We merely intend to present to our friends, more perhaps for their amusement than their edification, a few anti-

* For every information relating to the general, medical, and statistical history of the present condition of public charity in France, comprising a detailed account of all establishments destined for the sick, the aged, and the infirm, for children, and for lunatics, we refer our readers to the very excellent work of Dr. DAVID JOHNSON, which has been recently published.—ED.

phrenological truths, which the phrenologists will quickly refute, or the inevitable conclusion must be, that their science *has* been, but *is* no more. The supporters of the "science," warmed by enthusiasm into a very dangerous degree of confidence, exclaim, "Assail our facts, and we are undone!" Mr. Stone takes them at their word, and, in our opinion, very clearly shows that their facts are fables. We shall devote a page or two to the inferences Mr. Stone has derived from his investigations; then briefly state our opinion of Mr. Combe's "Answer" and Mr. Stone's "Rejoinder;" and conclude a very brief article by selecting a few of the obstacles which Dr. Wayte has thrown in the path of the phrenologists, in his argumentative and very temperate pamphlet.

The following are the inquiries which Mr. Stone has instituted:

"1. Does the phrenological developement of the murderer Burke correspond with his acknowledged character?

"2. Does the phrenological developement of his infamous accomplice Hare correspond with his acknowledged character?

"3. Is it possible to distinguish the crania of murderers from other crania, by the phrenological indications attributed to them?

"4. Do the most notorious thieves possess the organ of acquisitiveness larger, or that of conscientiousness smaller, than individuals of exemplary character?"

The organ of destructiveness in Burke has been called *large*. Mr. Stone inquires into the correctness of this report, and compares it, both in its absolute and relative size, with the same organ in two series of crania. With fifty crania, principally British, collected by Sir William Hamilton, and with the same number collected by Dr. Spurzheim, which are now in the Edinburgh Museum. The organ of amativeness was also particularly attended to, as Burke manifested the propensity attributed to it in an excessive degree. The counter-phrenological propositions which result from Mr. Stone's examination are:

"1st. The organ of destructiveness in Burke is absolutely and relatively *below* the average size, whilst benevolence and conscientiousness are absolutely and relatively *above* the average size.

"2d. The cerebellum* in Burke was also below the average size."

The counter-phrenological propositions deduced from the case of Hare are:

"1st. The organ of destructiveness is, in this atrocious murderer, *not above* the average size.

* The supposed seat of the organ of amativeness.

"2d. Many individuals of exemplary character, at the same time that they possess the organ of destructiveness larger than Hare, exhibit a greater deficiency in the alleged organs of benevolence and conscientiousness."

From the measurements of the crania of sixteen murderers, Mr. Stone derives the following reply to the third question:

"1st. The most atrocious murderers not only fail to possess a large endowment of the alleged organ of destructiveness, but have it very frequently, both *absolutely* and *relatively*, below the average size.

"2d. The most cruel and horrid murderers frequently possess a high developement of the pretended organs of the moral sentiments, particularly those of benevolence and conscientiousness.

"3d. Murderers do not possess a less developement of the supposed intellectual organs, nor a greater developement of those to which the animal propensities are referred, than individuals of high intellectual and moral character."

To determine the fourth question, Mr. Stone has taken measurements of the organs of acquisitiveness and conscientiousness, and at the same time the general size of the head, in an unselected class of individuals, English, Scotch, and Irish; and compared these with similar measurements from the heads of all the most notorious thieves in the Edinburgh jail and bridewell. The counter-phrenological proposition deduced is, "that the organ of acquisitiveness is often absolutely and relatively *less*, and that of conscientiousness absolutely and relatively *larger*, in the most notorious thieves, than in individuals of exemplary character."

The only comment Mr. Stone conceives it necessary to make on these deductions is expressed by Mr. Combe, who, in speaking of the truth or falsehood of phrenology, remarks: "If two individuals were found to possess a larger developement of acquisitiveness; but if in the one conscientiousness was very large, and in the other very small, and we were told that the one was a thief, and the other an honest man, how complete would the refutation be, if the one possessing the larger conscientiousness were found to be the rogue."*

It is proper to add, that the facts which led Mr. Stone to the conclusions above stated were taken without selection. In living individuals, the measurements of the first who presented themselves were taken, and the same plan was adopted with the crania; nor did Mr. S., in a single instance, reject the measurement of a person or cranium,

* Phrenological Transactions, p. 323.

because it did not appear to correspond with anti-phrenological evidence.

Mr. Stone's "Observations" have been followed by Mr. Combe's "Answer," and next comes Mr. Stone's "Rejoinder." We may be allowed, *en passant,* to return our thanks to Mr. Stone for *both* these last pamphlets. The two were tacked together, and forwarded to us, "with Mr. Stone's compliments." We state this circumstance for the purpose of showing that Mr. S., at least, conducts the combat fairly and fearlessly; for he himself furnishes us with the arguments of his opponent.

We have read, deliberately and attentively, both the Answer and the Rejoinder, and, as far as we are capable of judging, Mr. Stone clearly shews that Mr. Combe has not succeeded in his attempt to prove the fallacy of the anti-phrenological deductions which we have just extracted from the "Observations on the Phrenological Developement of Burke, &c." One passage from Mr. Stone we cannot forbear from giving:

"Mr. Combe states that Mr. Stone's prior pamphlet, his boasted 'Evidences against Phrenology,' has been dissected by an *able* writer in the London Medical and Surgical Journal, and wonders the newspaper editors of this city (Edinburgh) were not aware of this circumstance. Mr. Combe thus appears to sanction the statements of that writer; but will *he* become responsible for their insinuated veracity? Will *he* reiterate, on his *own* authority, the same accusations of 'misrepresentation,' 'erroneous quotation,' 'glaring interpolation?' &c. If so, I will answer him; but I will not, for a moment, think of noticing a scurrilous *anonymous* production, wherein I recognize neither the style of a gentleman nor the information of a scholar."

The "Anti-Phrenology" of Dr. Wayte has so recently appeared in the field, that the phrenologists have not yet had time to reply to it. We assure them that this little work is especially worthy of their attention; and, *when* they refute the various arguments it contains, we shall be among the first to do justice to their ingenuity. If they will condescend to accept our advice, they will not assail the man, but be contented with attacking his matter.

As we have determined to confine this extra-practical article within very moderate bounds, Dr. W. will excuse us if we select but one or two of the very satisfactory anti-phrenological doctrines with which his pages teem. Dr. Spurzheim says, "With respect to many individual parts, I have been certain of their *functions* for a long time, and

I could challenge any one to bring me an exception; but of *some others I will not speak so decidedly*."* To this remarkable confession of uncertainty in "the science" by *such* authority, Dr. Wayte very properly replies,

"Had phrenologists not delineated on the head any other organs than what they knew to a certainty, we might only be induced to think them rather dogmatical; but, when we find them depicting as exactly organs whose existence, and even nature, are doubted, then we have a legitimate right to question and distrust the accuracy and soundness of the whole science; for surely it evinces great imperfection to map out a brain into so many distinct faculties, name them definitively, and then be uncertain as to the reality of *many*. What should we think of a geographer, who, having clearly delineated the several places of a newly discovered country, afterwards informs us in his work that he could not speak decidedly as to some of them? Would his chart be worth a groat?"

Dr. W. now attacks the key-stone of phrenology: he shews, by the clearest arguments, that the principal tenet of the phrenologists, namely, plurality of organs, is founded upon the loosest reasoning, and that it has no foundation either in anatomy, physiology, pathology, or analogy. Another tenet has been broached, equally hypothetical.

"They (the phrenologists) assert that the brain is double, yielding a duplicate of each organ, inferred by their favorite mode of reasoning, from their being two eyes, two ears, and likewise from the nerves being given off in pairs. By this ingenious device, they in some measure overcome an argument which must otherwise completely refute their system: (to wit) that considerable portions of brain may be lost from injury, without any apparent diminution of intellect upon recovery; and their explanation of this is that the *corresponding organs* on the opposite side remain sound. There are, however, no solid arguments in favor of a double function of the brain, nor even of a perfectly double structure. True it is that the brain *apparently* consists of two halves; that it sends off from its base the nerves in pairs, one to each eye, to each ear, and so on; but it should be particularly borne in mind that there is an union or commissure between the two halves, and that their function is single. The motion of both eyes is quite synchronous, and their vision one: we have no double hearing, smelling, or tasting; and it is not because one eye may be lost, or one sense of hearing gone, or one limb removed, that we are to infer a double structure and action of the brain; for the analogy, to be just, should extend further. Thus every one knows that man can exist and officiate with only one eye, one ear, or one arm, &c.; and, since phrenologists inform us that, when one or *more*

* Dr. Spurzheim's Third Lecture, Lancet, vol. vii.

organs on either side of the brain are lost from injury, their *fellows* supply the defect, they ought, upon this principle, to make him exist, and perform all intellectual and animal functions, with only one *half of his brain;* when they can demonstrate this by showing me the man that under such a condition can 'live, and move, and have his being,' all my opposition shall cease."

We can follow Dr. Wayte no further in his very able and temperate refutation of phrenological fancies. We must conclude by observing, that the phrenologists have no right to expect others to agree with them until they agree with themselves. At present the advocates of the doctrine are at variance upon some of the most essential points of their creed; and, as a proof of the degree of confidence which is to be placed upon the practical application of the art, we may remind our readers of an anecdote mentioned by Dr. Burrows. Dr. Gall was requested to say, from the organs exhibited on a certain bust, what was the predominant propensity or faculty of the individual. He pronounced the original must be a great poet. His attention was directed to a second bust: he declared the latter to be that of a great mathematician. The first was the bust of Troughton, the eminent mathematician; and the second that of Sir Walter Scott! Mr. Chantrey also exhibited to Dr. Gall drawings of numerous heads. The cranioscopist selected one, whose ample cerebral developement gave a sure index of vast talent: it was a fac-simile of the head of the Earl of P-mf—t! Very recently, too, a most zealous and well-practised phrenologist discovered, from his examination of the head of a woman who had been hanged the day before for the murder of her child, that she must have had a *great attachment* for her children!

Let it not be imagined that we wish to insinuate the phrenologists wilfully attempt to deceive the public: they are enthusiasts, and deceive themselves. Certainly.

---

*An Essay upon the Treatment of the Deep and Excavated Ulcer; with Cases.* By Richard Anthony Stafford, Member of the Royal College of Surgeons, and lately House-Surgeon to St. Bartholomew's Hospital.—8vo. pp. 72. Longman, 1829.

Every practical surgeon must be aware that when an ulcer has eaten deeply into the substance of the flesh, and has thus formed a cavity, that the process of healing is extremely slow. Among such ulcers may be enumerated the open bubo which has burrowed deeply, and excavations which have been formed by the rapid destruction of the part by sloughing phagedena, or from any other cause; old indolent ulcers of the legs; indolent and deep ulcers arising from scrofula; in short, such as are situated in any part of the body where considerable substance has been lost. Hitherto cases of this sort have been far from obedient to any of the various plans of treatment that have been usually adopted. Mr. Stafford, amongst others, has perceived how much room there was for improvement in our practice in such cases, and he has exerted his ingenuity to devise a more successful and less tedious process of cure. The treatment he proposes has the merit of simplicity and perfect safety.

"It consists in pouring into the excavation melted wax, of an extremely adhesive quality, and just at that temperature when it is on the point of cooling, and will immediately become solid in the wound. In this manner the under surface of the wax, when cold, comes in close contact with the general surface of the ulcer, and the whole excavation is filled by it. Before employing it, however, it is necessary that one or two precautions should be taken: first, in order to clean the sore, as much of the pus as possible which rests upon it should be absorbed by dry lint; and secondly, in order to avoid burning the patient, the wax should be at that point of heat which is called by chandlers *setting;* that is, a portion of it should cling to the sides of the vessel in which it was melted, and the rest should begin to thicken, and have somewhat of an opaque appearance. In this state it will not be at much more than blood heat, and it can be used with perfect safety. It is advisable, however, even when so far cooled, that a brush be dipped into it, and that the wax be allowed to drop from that into the sore. After the wax becomes perfectly solid in the ulcer, a strip or two of adhesive plaster may be applied over it, to keep it in its situation; when it may be left until it requires to be dressed again, which will be on the third day after its application. By pursuing this method of treatment, it will be found that healthy granulations will be produced, and appear upon the whole surface of the sore; that it will contract; and that the healing process will proceed very rapidly."* (P. 4.)

Mr. Stafford does not imagine that the good effects which result from this treatment are entirely referable either to the complete exclusion of air from the affected part, or to the uniform pressure kept up upon the ulcer. He believes that the wax, being a foreign substance lodged in the excavation of the ulcer, becomes subject to the laws by which extraneous matter is expelled from the body. According to the idea he has formed of this process, the part immediately above the foreign body ulcerates, and from beneath

* The wax alluded to may be procured at Field and Son's, Wigmore street. It consists of four parts white wax, and one part Venice turpentine.

it a new growth is established, which pushes it on until it arrives at the surface.

"Let us suppose, for example, that a piece of dead bone, or foreign matter, is lodged in the fleshy part of the thigh: how will nature throw it off? First, the part above it will ulcerate; and, secondly, a new growth will take place from beneath it; whereby not only will the bone be forced on, and thus, by the pressure it makes above, the ulceration will be continued, but the cavity which must necessarily be made by it will in this manner be filled up." (P. 9.)

When an extraneous body is buried in the substance of the flesh, the process by which it is expelled cannot be seen; but when the death of a part (which consequently becomes a foreign body) takes place on the surface, we may see the progress of nature in the operation.

"The mortified part is first separated from the living by ulceration, which forms a kind of *cordon sanitaire* all around it, gradually extending beneath it, from the edges to the centre; and, as fast as the separation takes place, granulations spring up from the interior of the excavation, and, by the time the whole process is completed, the cavity is nearly filled by them. In this manner the mortified part is protruded by the granulations, and expelled from the situation it previously occupied." (P. 10.)

Mr. Hunter slightly alludes to this fact.* Mr. Stafford grounds the basis of his treatment upon the second process, viz. the springing up of granulations upon the surface of the ulcer, to throw off the extraneous matter resting upon it.

"The wax may be considered to be the foreign body, or mortified part, separated from, but in close contact with, the ulcer; and, as it is one of nature's laws to throw off extraneous matter, granulations are engendered upon its whole surface to effect this purpose; and thus a natural process is imitated. That this is the case may be inferred from the solid wax, after a time, being found partly thrust out of the cavity; and, if removed, it cannot be made to adapt itself as before." (P. 12.)

Having endeavoured to explain what he conceives to be the process of the healing of an ulcer, when filled with the wax, Mr. Stafford proceeds to point out the process by which it heals; the superiority of this plan of treatment; and the cases in which it will be advantageous. The progress by which a sore heals must in some measure depend upon its size, the health of the patient, &c. The stages by which the reparative process is usually carried on, when treated with the wax, is as follows:

"On the removal of the first dressing, the sore generally presents

* Hunter on the Blood, &c. vol. ii. p. 361.

a cleaner surface, being more reddened; and sometimes, even in the early stages, granulations are distinguishable. After the second dressing they are commonly spread over the whole surface of the sore; on the third, they partly fill up the cavity, which is much contracted; and on the fourth, it appears still less; and so on until it is completely closed, and then the skinning process commences. During the course of healing, likewise, it may be observed that the granulations are smaller, more compact, and more florid. The cicatrix also presents a more even surface; it is of a firmer texture, less tender, and does not appear so likely to break out again as the scars of those ulcers which have not been treated according to this plan." (P. 14.)

By this plan Mr. S. assures us that the sore is healed in one third of the time usually occupied, and with much greater certainty than where the common methods are employed. That it succeeds where no other remedy will, is shewn by the cases. The pain when the wax is upon the sore is so trifling, that many patients have not only been unconscious of its presence, but even of the existence of the sore itself. The treatment is applicable to ulcers of any depth, from whatever cause they may arise. When the ulceration has been extending, its progress has been immediately arrested, and it has shown a disposition to heal; and, when the sore has been connected with varicose veins of the legs, it has been attended with great advantage.

"Although there are very few species of ulcers where this method of treatment might not be successful, yet it offers peculiar benefit in many cases which before defied all other remedies: for instance, in sores situated over large arteries, where there is danger of the ulcerative process being continued into the vessel. Such cases are not uncommon; and, to my own knowledge, several patients have died in consequence of every application having proved ineffectual to stop its progress. In these instances it is customary to apply stimulating remedies; but every practitioner must have observed that such dressings have too often accelerated the catastrophe they have intended to ward off. Here, then, the use of the wax might be of singular advantage, by producing healthy granulations, and at the same time protecting the artery. The same plan of treatment might likewise be resorted to in all extensive sores, such as burns; and thus not only might they be made to heal more quickly, but they would likewise be shielded from objects around them. There are some species of ulcers, also, whose peculiar character it is to spread; for instance, herpetic sores, noli me tangere, and cancerous ulceration; and if the principle which I have pointed out as to the action which this plan of treatment induces in the ulcer be correct, it might possibly be of infinite service in these cases, in putting an end to, or at least stopping the progress of, the ravages of the disease; and more particularly when it is extending itself into parts so full of blood-vessels that there is reason to apprehend the death of the patient from hemorrhage. I have not had an opportunity of employing it in any of these cases, excepting in cancerous ulceration, where its effects in producing granulations was extroardinary; consequently I am unable to bring forward any facts to establish its utility, and therefore I merely offer these remarks as a suggestion." (P. 18.)

We are not to place such unlimited confidence in any local treatment as may induce us to neglect constitutional remedies, if the health be deranged.

We shall select two or three of the cases detailed by the author. It may be proper to observe, that the plan has been so successful, and in such a variety of instances, that Mr. Charles Phillips almost always resorts to it in the cases that occur in the St. Marylebone Infirmary.

Case I. *Ulcerated Legs.*—John Covill, æt. forty-nine, has had an extensive ulcer for twenty-five years. About ten years ago it healed for a short time, but it broke out again almost immediately, and has remained open ever since.

" The sore, as it now exists, occupies nearly the whole space between the calf and the ankle, and extends nearly all round the circumference of the leg. It is about one third of an inch in depth, of an excessively foul character; and the discharge issuing from it is extremely offensive. Applications of every description have been employed, without its showing the least disposition to heal or to change its character. Under these circumstances, and as the man had been crippled by it for many years, it was proposed that the limb should be amputated. The patient, however, would not consent to the operation." (P. 22 )

Upon the principles above explained, Mr. Stafford procured some wax, of as adhesive a quality as possible, melted it, and, as it cooled, poured it into the ulcer.

" The patient immediately expressed relief from its application, and it was left on the sore three days. On the fourth, when it was about to be removed, it was found to be slightly raised; and, on taking it away, the surface of the ulcer was seen, to the astonishment of every one, covered by granulations, and its depth was considerably diminished. These granulations were much smaller than those on healthy sores in general, being about the size of small shot; they were much more regularly disposed, and they were of a beautiful florid red colour.

" Feb. 17.—The sore was again dressed with the melted wax, and on the fourth day was removed. The wound still presented the same appearances as when last seen, excepting that about two thirds of the excavation was filled up. The application of the wax was continued.

"Feb. 21.—The granulations were equal with the surface of the leg; they still retained their diminished character and their florid hue.

"25.—The sore had healed one fourth of an inch all around its circumference, the granulations still having the same appearance.

"28.—The sore still less.

"March 2.—The sore is only half the size it was at first, and the healing process is proceeding very rapidly.

"About a fortnight from this time the man was discharged perfectly cured. The cicatrix was much firmer, and more regular than that of ulcers in general. The medical treatment was simply keeping the bowels regular." (P. 23.)

In the second case, a woman, æt. sixty-eight, had extensive ulcers, of considerable depth, on both legs, which broke out thirty years before, and had been open for the last six years. The sores were deep, painful, and of a most irritable character. This patient was under treatment seven weeks, and was then discharged cured. The cicatrices were firm and even, and she has remained well ever since. The medical treatment was simply occasional purgatives.

Thirteen similar instances are related, and as many as from 150 to 200 cases of ulcerated legs have been thus treated with success.

Eight cases of excavated buboes of the groin are next given, to show the efficacy of the practice in a different kind of ulceration. One will be sufficient as an example.

"Philip Quinlan, æt. twenty-five. Feb. 20, 1828.—Has an indolent excavated sore in the right groin, in consequence of bubo. It is in length about one inch and a half; in breadth rather more than halt an inch; and in depth two thirds of an inch. Its edges are ragged, and it is of an extremely foul character, being covered by a dirty yellow discharge. It has been in this state for more than six weeks, and no application hitherto used has changed its character, or disposed it to heal.

"The melted adhesive wax was applied, and the whole excavation filled with it. No pain was experienced; and, during the whole time it remained on, the sore was easier.

"23.—The wax was removed, and the sore was much improved. Granulations had sprung up from the bottom, and one third of the sore was filled by them. It was again used as before.

"25.—The cavity was filled about two thirds, and the ulcer was much contracted. The edges were less ragged, and the whole surface was covered with healthy florid granulations.

"28.—The excavation was entirely obliterated.

"March 2.—The size of the wound was much contracted, and the skinning process beginning to take place at the edges.

"5.—It is half-healed, much contracted, and its surface very regular.

" 8.—All but healed.

" 11.—Quite healed. The cicatrix is much firmer than common; it is smooth, and there are no ragged edges." (P. 45.)

Two cases of sloughing phagedena, and four cases of scrofulous ulcers, are also detailed; and in these instances Mr. Stafford's mode of treatment was applied with equal success. In two cases of cancerous ulceration in which it was employed, although both terminated fatally, yet granulations were rapidly produced, and thus the ulceration was for a time prevented from extending itself into large arteries.

This plan of treatment ought to be immediately tried upon an extensive scale in many of the numerous cases of obstinate chronic ulceration which occupy our hospitals, and other public institutions, for month after month, to the annoyance of the surgeon and the discredit of his art.

The zeal which Mr. Stafford evinces for the practical improvement of his profession is highly creditable to him.

